# Sieving carbons reconfigure non-graphitic carbons for practical sodium batteries

**DOI:** 10.1093/nsr/nwac126

**Published:** 2022-07-23

**Authors:** Xueliang Sun

**Affiliations:** Department of Mechanical and Materials Engineering, The University of Western Ontario, Canada

For alkali metal-ion batteries, carbon materials are always regarded as the most promising anodes for commercialization, as is seen in the history of lithium-ion batteries (LIBs). The success of graphite anodes in LIBs indicates the importance of low-potential charge/discharge plateaus (LPPs) for achieving high energy density [1]. As the counterpart to LIBs, sodium-ion batteries (SIBs) are showing great potential for large-scale energy-storage applications, owing to the natural abundance and low cost of sodium resources. In SIBs, some types of non-graphitic carbons are reported to deliver LPPs similar to those of graphite in LIBs [2]. However, challenged by the variable and complicated microstructure of non-graphitic carbons, strategies to produce and reversibly extend their LPPs remain unclear and this inevitably impedes the rational design of non-graphitic carbon anodes for practical SIBs.

**Figure 1.**
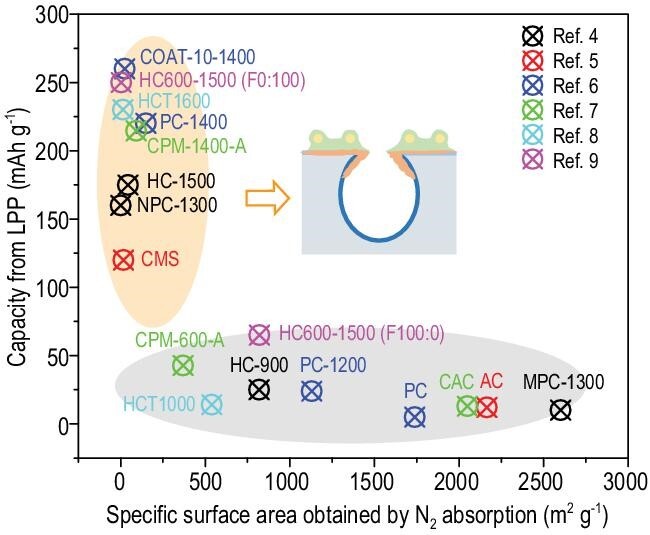
The correlation between the specific surface area obtained by N_2_ adsorption and the capacity from the LPP. Inset: the relative location of SEI to the nanopores. SEI is drawn as a laurel-green irregular shape.

Li *et al*. recently represented a conceptual advance for designing sieving carbons (SCs) by reconfiguring non-graphitic carbons, and achieved extensible and reversible LPPs of SC anodes in SIBs [3]. Similar to the lessons learned from the graphite anode in LIBs, the rational design of SCs have taken both the interfacial electrochemistry and electrode chemistry into careful consideration. Closed nanopores of non-graphitic carbons are believed to be crucial to the emergence of LPPs [4–9]. As vividly depicted in Fig. 1, if the specific surface area obtained by N_2_ adsorption is large, the capacity originating from the LPP is negligible. In contrast, if the specific surface area obtained by N_2_ adsorption is very low, indicating that its pore entrance diameter is <0.4 nm, a large portion of the reversible capacity comes from the LPP. Li *et al*. provided the scientifically important understanding on this interesting trend and revealed that the solid electrolyte interphase (SEI) hardly forms inside nanopores when the pore entrance is tightened (<0.4 nm), as evidenced by the *ex situ* small-angle X-ray scattering. In addition, with detailed studies, they explained the reason why the tightened pore entrance contributed to not only the significantly increased initial coulombic efficiency but also the production of LPPs.

**Figure 2.**
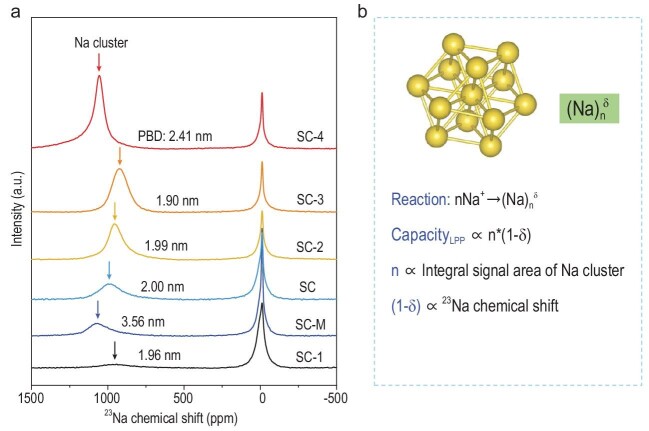
(a) *Ex situ*^23^Na ssNMR spectra of different SCs at 0.005 V for the first discharge. (b) The approximately quantitative relationship between the capacity from the LPP and the product of the ^23^Na chemical shift and integral signal area of the sodium cluster.

With unprecedented theoretical and spectroscopic results of SCs as the model anodes, Li *et al*. have systemically clarified the electrode chemistry, especially regarding the sodium-storage mechanism related to the LPPs. This has been a hotly debated topic for years. Li *et al*. have shown that bare sodium ions first adsorbed on the defective pore surface and aggregated to finally form the quasi-metallic sodium clusters inside closed nanopores. Since the LPP originates from the formation of sodium clusters, the capacity from LPP is proposed to be proportional to the total transferred charges of the sodium-clustering process—that is, the product of average transferred charge per clustered sodium atom and the number of clustered sodium ions in a unit mass of the SC. This can be obtained from ^23^Na ssNMR results, as shown in Fig. 2a and b. An approximately linear correlation between the specific surface area in SCs and the plateau capacity was revealed, leading to a record-high plateau capacity of 400 mAh g^–1^. However, a relatively small pore body diameter (<2.0 nm) was needed to avoid the formation of metallic sodium and guarantee the reversibility of the LPPs, which increases the cycling life of sieving carbon anodes. It is worth noting that soft carbons can also be used to prepare SCs with long and reversible LPPs; this is not limited to hard carbons as traditionally believed. It is the sieving pore entrance, together with highly developed nanopores, that produce and reversibly extend the LPPs of SC anodes. This is an innovative and fundamental understanding for non-graphitic carbon anodes, which could inspire more rational carbon designs for high-performance SIBs.

The tunable nanostructure of SCs indicates the possibility to further increase the capacity originating from the LPPs. Therefore, it is worthwhile to explore whether there is an upper limit of the reversible capacity for SC anodes in SIBs (must be <1166 mAh g^–1^, the theoretical capacity of sodium metal). Both careful theoretical simulations and pore structure designs are needed to provide a plausible explanation. Moreover, what if SC anodes are matched with solid electrolytes? Will the sodium-clustering process still occur and how will it be different from that in the liquid electrolytes? How will the nanoporous structure of SCs determine the physicochemical properties of sodium clusters?

To some extent, SCs represent the future of non-graphitic carbons in alkali metal-ion batteries. SCs are not only critical for accelerating the commercialization of SIBs as practical anodes, but also offering a tunable and ideal model carbon to understand the interactions between alkali metal ions and non-graphitic carbons.

***Conflict of interest statement.*** None declared.
